# Applying the Free Energy Principle to Complex Adaptive Systems

**DOI:** 10.3390/e24050689

**Published:** 2022-05-13

**Authors:** Paul B. Badcock, Maxwell J. D. Ramstead, Zahra Sheikhbahaee, Axel Constant

**Affiliations:** 1 Centre for Youth Mental Health, The University of Melbourne, Melbourne, VIC 3010, Australia; 2 Orygen, Parkville, VIC 3052, Australia; 3 VERSES Research Lab and the Spatial Web Foundation, Los Angeles, CA 90016, USA; maxwell.d.ramstead@gmail.com; 4 Wellcome Centre for Human Neuroimaging, University College London, London WC1E 6BT, UK; 5 David R. Cheriton School of Computer Science, University of Waterloo, Waterloo, ON N2L 3G1, Canada; zsheikhb@uwaterloo.ca; 6 Charles Perkins Centre, The University of Sydney, John Hopkins Drive, Camperdown, NSW 2006, Australia; axel.constant.pruvost@gmail.com

The free energy principle (FEP) is a formulation of the adaptive, belief-driven behaviour of self-organizing systems that gained prominence in the early 2000s as a unified model of the brain [1,2]. Since then, the theory has been applied to a wide range of biotic phenomena, extending from single cells and flora [3,4], the emergence of life and evolutionary dynamics [5,6], and to the biosphere itself [7]. For our part, we have previously proposed that the FEP can be integrated with Tinbergen’s seminal four questions in biology to furnish a multiscale ontology of living systems [8]. We have also explored more specific applications, e.g., to the evolution and development of human phenotypes [9,10,11], socio-cultural cognition, behaviour, and learning [12,13], as well as the dynamic construction of environmental niches by their denizens [14,15].

Despite such contributions, the capacity of the FEP to extend beyond the human brain and behaviour, and to explain living systems more generally, has only begun to be explored. This begs the following questions: Can the FEP be applied to any organism? Does it allow us to explain the dynamics of all living systems, including large-scale social behaviour? Does the FEP provide a formal, empirically tractable theory of any complex adaptive system, living or not? With such questions in mind, the aim of this Special Issue was to showcase the breadth of the FEP as a unified theory of complex adaptive systems, biological or otherwise. Instead of concentrating on the human brain and behaviour, we welcomed contributions that applied the FEP to other complex adaptive systems, with the hope of exemplifying the extent of its explanatory scope.

For the uninitiated, it is worth briefly outlining what the FEP is. Variational free energy refers to an information theoretic quantity that places an upper limit on the entropy of a system’s observations, relative to a generative model instantiated by an agent. (In this context, entropy is defined as the time-average of ‘surprise’ or the negative log probability of the agent’s sensory data.) Generative models harness probabilistic mappings from hidden causes in the environment to observed consequences (i.e., sensory data), and state transitions inherent to the environment [2]. Under the FEP, an organism is modelled as implicitly ‘expecting’ to find itself within a limited range of phenotypic states; as such, deviations from these states elicit a type of ‘phenotypic surprise’ (i.e., the deviation between actual and phenotypically preferred states). Consequently, organisms remain alive by acting in ways that minimize this type of surprise (e.g., a fish avoiding the ‘state’ of being out of water). In other words, and more heuristically, free energy scores the discrepancy between desired and sensed data; and the FEP states that the imperative of all self-organizing systems is to keep this discrepancy at bay by bringing about preferred observations via action (see Figure 1).

There are two main ways for a self-organizing system to minimize free energy. The first is by changing its perception of the world. Previously, this has been explored through reference to human neural processing. The FEP appeals to a view of the brain as a hierarchical “inference machine”, which instantiates a hierarchy of hypotheses about the world (i.e., a generative model) that enables an organism to minimize free energy (and therefore keep entropic dissipation at bay, at least locally) by reducing discrepancies between incoming sensory inputs and top-down predictions (i.e., prediction errors) [2]. Neurobiologically xpectations about sensory data are thought to be encoded by deep pyramidal cells (i.e., representation units) at every level of the cortical hierarchy, which carry predictions downward to suppress errors at the level below, whereas prediction errors themselves are encoded by superficial pyramidal cells and are carried forward to revise expectations at the level above [16]. The relative influence of ascending (error) and descending (representation) signals is weighted by their inverse variance or *precision* (e.g., a high precision on ascending error signals lowers confidence in descending predictions), which is mediated by neuromodulation and reflected psychologically by attentional selection and sensory attenuation. In short, the recursive neural dynamics described here enable us to minimise free energy (resp. prediction error) by updating our internal models (i.e., perception).

Second, an organism can reduce surprise directly by acting upon the world in order to fulfill its expectations and generate unsurprising sensations. This process of ‘active inference’ describes how an organism reduces free energy through self-fulfilling cycles of action and perception [17]. Active inference models implement action selection as the minimization of *expected free energy*, which is the free energy expected under beliefs about possible courses of action, or policies. By selecting actions that are expected to minimize free energy, the organism can maintain itself within preferred, phenotypically unsurprising states. Thus, survival mandates that an organism must not only reduce free energy from moment to moment; it must also reduce the expected free energy associated with the future outcomes of action [18,19].

Having briefly outlined the rudiments of the FEP, let us turn briefly to complex adaptive systems (CAS). This concept is synonymous with complexity science and has its roots in evolutionary systems theory, which assumes a dynamic, inextricable relationship between generalised selection and self-organization [11]. Broadly speaking, a CAS refers to any multi-component, self-organising system that adapts to it environment through an autonomous process of selection, which recruits the outcomes of localised interactions between its components to select a subset of those components for replication and enhancement [20]. Holland [21] describes four key features of CAS: they consist of large numbers of components that interact by sending and receiving signals (i.e., *parallelism*); the actions of their components depend upon the signals they receive (i.e., *conditional action*); groups of rules can form subroutines that can be combined to deal with environmental novelties (i.e., *modularity*); and the components of the system change over time to adapt to the environment and improve performance (i.e., *adaptation*). Applications of the CAS framework have proliferated across the physical, human and computer sciences, but there is not the scope to survey this literature here. However, to pre-empt the papers to follow, we would note that this framework has already been applied to precisely the same systems that are the foci of our contributors–ranging from metabolic and cellular processes, e.g., [22,23,24,25,26]; to the brain and social processes, e.g., [26,27,28,29,30]; and to artificial intelligence and robotics, e.g., [31,32,33]. The articles presented in our Special Issue build upon such literature by illustrating how the FEP can afford fresh insights into the dynamics of CAS.

Three of the contributions to our Special Issue leverage the FEP to cast new light on processes intrinsic to biological agents. In *Cancer Niches and their Kikuchi Free Energy*, Sajid, Convertino et al. [34] examine cancer morphogenetic fields as undesirable stable attractors in the complex dynamics of homeostasis, self-renewal and differentiation, which contributes to their deviation from regular autopoietic homeostasis (the internal molecular dynamics that regulate the production and regeneration of a system’s components). Sajid, Convertino et al. offer a computational model in silico to study communication and information processing at a population level of cancer cell networks within their environment in vivo. By deploying the Kikuchi free energy approximation, which is a generalisation of the Bethe free energy for computing beliefs over large sets of cell clusters, they account for higher-order interactions and phase transitions between clusters of healthy and oncogenic cells. Here, cancer niche construction can be construed as a Bayes-optimal process for the transmission of information across different levels of cellular networks due to its tendency to minimize the overall Kikuchi free energy over the whole system. Their findings suggest that three distinct cancer trajectories–namely, proliferation (local growth), metastasis and apoptosis–can emerge from the natural evolution of the state function (i.e., free energy) in biological systems. These findings have important implications for our understanding and study of cancer cell growth and apoptosis.

Next, Parr describes how biochemical networks in adaptive biological systems can be recast in terms of an inferential message passing scheme that involves the gradient descent on variational free energy towards the least surprising states, based on the organism’s implicit (generative) model of these states. In *Message Passing and Metabolism* [35], he points out that the biochemical regulation of metabolic processes relies on sparse interactions (message-passing) between coupled reactions, with enzymes creating conditional dependencies between reactants. He then extrapolates the *law of mass action* (the rate of chemical reaction and the concentrations of reactants involved in this process) and the *Michaelis–Menten kinetics* (which approximates the dynamics of irreversible enzymatic reactions) from the FEP. Assuming the existence of a causal structure in biochemical (metabolic) networks, one can build the sparse message passing scheme to capture the independence of substrates and products, conditioned upon the enzyme and enzyme-substrate complex within such networks. The temporal evolution of the categorical probability of each state within this system can be described by a chemical master equation that takes into account sparse network interactions. Parr describes how the steady state distribution of these dynamics can be recast as a generative model, which suggests that the biochemistry that underlies metabolism follows an inferential message-passing scheme that seeks to minimise free energy. An important extension of Parr’s model is that metabolic disorder can emerge when an enzymatic disconnection by thiamine depletion interrupts message passing and incites aberrant prior beliefs, which gives rise to false (biochemical) inference.

The third contribution follows more traditional applications of the FEP by accounting for conscious, first-person experience. In *The Radically Embodied Conscious Cybernetic Bayesian Brain*, Safron [36] proposes models of embodied conscious agency based on the FEP, extending the Integrated World Modelling Theory of consciousness proposed elsewhere [37] to explicitly account for aspects of intentional actions and agentic experiences. According to the radically embodied account on offer, what we call attention and imagination emerge from the (sometimes liminal) activity of multimodal, action-oriented body maps and representations, realized as neural attractors in the form of ‘embodied self-models’ (ESMs), which conform to the FEP as cybernetic controllers. When functioning online, ESMs allow for overt interactions with affordances, or structured possibilities for environmental interactions. However, Safron suggests subthreshold activations of such ‘quasi-homuncular’ ESMs also underwrite our (affordance-structured) covert abilities to imagine and pursue courses of action, as well as our ability to intentionally deploy attentional resources. Thus, even seemingly abstract representational capacities may be grounded in twin capacities for embodied action and counterfactual explorations of the world. Safron then applies this radically embodied perspective to core aspects of conscious experience. He attempts to chart a middle way between perspectives in the representation wars in cognitive science, describing brains as hybrid machine learning architectures capable of supporting both symbolic and sub-symbolic processes for 4E agents (where cognition is thought to be *embodied*, *embedded*, *enacted* and *extended*). Safron’s perspective is ecumenical, deploying information-theoretic constructs and representationalist concepts that would be rejected by hard-line proponents of both 4E cognition and more Cartesian (representationalist) approaches. For example, to account for information flow in mammalian brains, Safron deploys constructs that are typically rejected by 4E theorists, such as Cartesian theatres and quasi-homunculi. However, he does so from a radically embodied perspective, suggesting that such a “strange inversion of reasoning” follows from principles of cognitive development and computational neuroscience.

Unlike the authors above, Goekoop and de Kleijn look beyond the phenotype to consider how the FEP might apply to groups. In *Permutation Entropy as a Universal Disorder Criterion* [38], they argue that living systems can be described as hierarchical problem-solving machines that embody predictive models of their econiches, called a goal hierarchy, which incorporates a set of lower-order econiches (goals) and corresponding subniches (subgoals) that the system needs to pursue in order to achieve the global econiche (goal) represented at the top of the hierarchy. Using this scheme to frame the rest of their argument, they concentrate on stress responses in organisms, dyads and collectives. Equating stress with free energy or ‘prediction error’, and stress responses with ‘action’, they suggest that as free energy increases, there is a progressive collapse of (allostatic) hierarchical control, eventually resulting in disordered states characterised by behavioural shifts from long-term goal-directed behaviour (e.g., reproductive success) towards short-term goals and habitual behaviours concentrated on self-preservation (e.g., survival). After introducing permutation entropy as a universal measure of disordered states across such systems, they briefly describe how their model can be used to explain disorder at an individual level, before progressing to the transmission of disorder through interpersonal interactions, and concluding with a brief discussion of population-level dynamics.

The idea that the FEP can be extended to social systems is also taken up by Kaufmann and colleagues. In *An Active Inference Model of Collective Intelligence* [39], the authors propose an active inference model of alignment, describing the manner in which within-scale local interactions (e.g., individual agents’ behaviors) can align with cross-scale global phenomena (e.g., collective behavior) in multi-scale systems. In so doing, they offer a principled, agent-based model that has the potential to function as a workbench to simulate collective intelligence as an emergent phenomenon, across many scales. Although one obvious target for this modelling approach would be human behavior as an emergent phenomenon that ties physiological, cognitive and cultural dynamics, nothing, in principle, limits the application of Kaufmann and colleagues’ model to human phenomena.

In another paper that illustrates the broad scope of multiscale thinking under active inference, Jesse Hoey, in *Equality and Freedom as Uncertainty in Groups* [40], shows how agents attempting to align with other group members leads to a quasi-equilibrium, or “sweet spot”, at which the group free energy is minimal and the agent’s predictive capacity of higher order parameters, such as those attributed to the group, matches the group’s capacity to predict an agent’s behavior. Hoey further discusses two intriguing trade-offs. Higher agent model complexity leads to lower individual learning capacity with respect to the complexity of the group, resulting in agents who are hobbled in the pursuit of their own ends, but in a group that is more diverse, innovative, and open to change. On the other hand, lower agent model complexity allows the expression of individual preference towards the group, but the group becomes more homogeneous, secure, and closed, as otherwise the pro-social behaviours of individual agents would be hampered. Hoey suggests that such emergent social dynamics provides insights into concepts such as freedom and equality in society, which correspond to changes in model uncertainties and complexities. Oscillation between radical freedom, where no cooperation is possible, and radical equality, where no discovery is possible, is an emergent phenomenon characteristic of Western society; akin to what Karl Marx called historical materialism, which according to many is the main driver of history itself. Could Hoey′s findings initiate research on an active inference model of history as an emergent phenomenon of human societies?

So far, we have considered a range of applications of the FEP to living systems. However, active inference–the process theory derived from the FEP–is increasingly being applied to machine intelligence in practical settings. Use cases in robotics provide an exciting opportunity to test the applicability of active inference to implement sensory processes and motor control in real time. In their review for our Special Issue, *How Active Inference Could Help Revolutionise Robotics*, Da Costa and colleagues [41] examine the usefulness of active inference for several core problems in robotics, such as state estimation in artificial perception, motor control, learning, safety and explainability. They argue that active inference may help advance robotics due to several of its core features: it enables explainable artificial intelligence in a manner that operates in situations involving uncertainty, volatility, and noise. This is especially relevant for human-centric applications, such as human–robot interaction and healthcare.

In closing, it is worth recognising that the majority of submissions presented herein focus chiefly on human systems, despite our call for more wide-ranging applications. Nevertheless, it should be clear that the authors’ proposals can be readily extended to other complex adaptive systems, including biological dynamics intrinsic to other lifeforms [34,35,36], collective, group-level behaviour [39,40], and even non-living systems [41]. Taken together, we hope that the collection of papers presented in our Special Issue motivate others to consider how the FEP might be gainfully applied to their own systems of interest, living or otherwise.

## Figures and Tables

**Figure 1:**
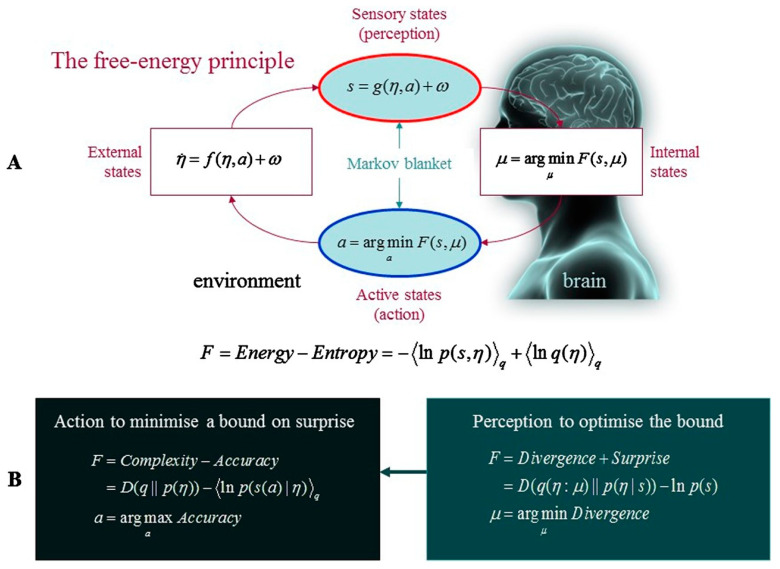
**The free energy principle**. (**A**) Schematic of the quantities that characterise free energy, including a system’s internal states, *μ* (e.g., a brain), and quantities that describe the system’s exchanges with the environment; specifically, its sensory input, *s* = *g*(*η*,*a*) + *ω*, and actions, *a*, which alter the ways in which the system samples its environment. Environmental states are further defined by equations of motion, $\dot{\eta }$ *= f(η,a) + ω*, which describe the dynamics of (hidden) states extraneous to the system, *η*, whereas *ω* refers to random fluctuations. Under this scheme, internal states and action operate synergistically to reduce free energy, which reflects a function of sensory input and the probabilistic representation (variational density), *q*(*η*:*μ*), that internal states encode. Note that external and internal states are statistically separated by a Markov blanket, which possesses both ‘sensory’ and ‘active’ states. Internal states are influenced by, but cannot affect, sensory states, whereas external states are influenced by, but cannot affect, active states, creating a conditional independence between the system and its environment. (**B**) Alternative equations that describe the minimisation of free energy. With respect to action, free energy can only be suppressed by the system’s selective sampling of (predicted) sensory input, which increases the accuracy of its predictions. On the other hand, optimising internal states minimises divergence by making the representation an approximate conditional density on the hidden causes of sensory input. This optimisation reduces the free energy bound on surprise, which means that action allows the system to avoid surprising sensations. Reproduced from [8].
